# The impact of serum ferritin on overall survival following resection in patients with intrahepatic cholangiocarcinoma

**DOI:** 10.1007/s00423-025-03737-1

**Published:** 2025-05-21

**Authors:** Laura Schwenk, Carlos Wolf, Felix Dondorf, Oliver Rohland, Aladdin Ali-Deeb, Utz Settmacher, Falk Rauchfuß

**Affiliations:** 1 Department of General, Visceral and Vascular Surgery, Jena University Hospital, Am Klinikum 1, 07740 Jena, Germany; 2 Comprehensive Cancer Center Central Germany– Campus Jena, Jena, Germany; 3 Interdisciplinary Center for Clinical Research (IZKF), Jena University Hospital, Jena, Germany

**Keywords:** Intrahepatic cholangocarcinoma, Serum ferritin, Liver resection, Overall survival

## Abstract

**Purpose:**

The global incidence of intrahepatic cholangiocarcinoma is increasing. Surgical resection remains the gold standard treatment. However, the long-term prognosis remains dismal. The role of serum ferritin in malignant diseases has not been fully elucidated. This study aimed to evaluate the relationship between preoperative serum ferritin levels and patient outcomes.

**Methods:**

In our retrospective study, we analyzed data from 95 patients who underwent liver resection for intrahepatic cholangiocarcinoma at Jena University Hospital between 2009 and 2023. Comprehensive clinical and pathological data, along with the correlation between Serum ferritin and clinicopathological parameters, were systematically analyzed and compared. Survival rates were determined using the Kaplan-Meier method.

**Results:**

The optimal preoperative serum ferritin cut-off value for overall survival was 303.1 µg/L, with an area under the curve of 0.697 (95% CI (0.592–0.801; *P* < 0.001). The 1-, 3-, and 5-year survival rates were 74.7%, 50.5%, and 43.2%, respectively. Patients with elevated preoperative SF levels demonstrated significantly worse overall survival compared to the low SF group (50.9% vs. 4.5%; *P* < 0.001). SF had a significant impact on recurrence rates (*P* < 0.001). The overall recurrence rate in the high-SF group was 67,3%, compared to 43,5% in the low-SF group.

**Conclusion:**

Elevated preoperative serum ferritin levels are associated with significantly worse overall and recurrence-free survival in patients with intrahepatic cholangiocarcinoma. Serum ferritin could serve as a valuable adjunct to the tumor marker CA 19 − 9.

## Introduction

The incidence of intrahepatic cholangiocarcinoma (iCCA) has been increasing globally in recent years [1, 2]. ICCA is the second most common primary liver cancer after hepatocellular carcinoma, accounting for approximately 10–15% of all primary liver malignancies [2]. Diagnosis often occurs at an advanced stage, leaving palliative chemotherapy as the only treatment option, with dismal survival rates below 10% [3]. Surgical resection remains the gold standard for treatment, with reported overall survival rates of up to 34% [4, 5]. Identifying new prognostic factors that influence survival in patients with iCCA is therefore of increasing importance. However, laboratory-based surrogate markers remain insufficiently explored and evaluated to date.

The glycoprotein ferritin was first discovered in 1937 by Victor Laufberger, who isolated a novel protein from the spleen of horses [6]. Years later, ferritin was also detected in human serum. Ferritin is primarily known for its role in iron metabolism as an iron storage protein. The exact secretion pathway of SF remains unclear, although hepatocytes, macrophages, and Kupffer cells are known to secrete ferritin. Elevated SF levels are not only observed in cases of iron overload but also in infections, organ dysfunction, liver diseases, and chronic illnesses. Its exact pathophysiological function is still not fully understood. The role of SF in malignancies has been increasingly investigated and debated in recent years. The association of serum ferritin with malignancies and its potential role as a useful tumor marker was first postulated in 1977 by Hazard and Drysdale [7]. Since then, numerous studies have demonstrated a correlation between elevated SF levels and malignancies [8–12]. However, no studies to date have investigated the impact of SF on iCCA. Therefore, the aim of our study was to evaluate the relationship between preoperative serum ferritin levels and patient outcomes.

## Materials and methods

The data of patients who underwent liver resection for iCCA at the University Hospital in Jena between 2009 and 2023 were evaluated. The following parameters were analyzed: preoperative SF levels, overall survival (OS), 3-year and 5-year survival rates, recurrence rates, and tumor-specific information such as tumor staging (TNM 8th edition), size and differentiation.

Tumor markers and general patient data, based on clinical, surgical, and pathological findings, were collected from the hospital’s SAP database (SAP Global Corporate Affairs, Walldorf, Germany). Due to incomplete pathological results, exact data regarding the tumor staging (T-stage, N-stage) and tumor size are unavailable for a very small number of patients. Selected patient data are presented as means and ranges. Preoperative serum ferritin levels were measured one to three days prior to surgery. The cut-off value for CA 19 − 9 was set at 37 U/mL.

### Statistical analysis

The optimal cut-off value for preoperative serum ferritin (SF) was determined using the Receiver Operating Characteristic (ROC) curve. The point on the ROC curve with both the maximum sensitivity and specificity was selected as the best cut-off value to define elevated SF levels.

Survival curves were generated using the Kaplan–Meier method, and group differences in overall survival were assessed using the log-rank test. To identify independent prognostic factors for overall survival, a multivariate Cox proportional hazards regression analysis was performed. Variables included in the model were selected based on clinical relevance and univariate analysis results. The final model comprised preoperative serum ferritin, T-stage, G-stage, tumor size, N-stage, albumin, and CA19-9. Preoperative SF levels were dichotomized using the ROC-derived cut-off of 303.1 µg/L, with levels > 303.1 µg/L classified as elevated. Hazard ratios (HRs) with 95% confidence intervals (CIs) and corresponding *p*-values were calculated. The proportional hazards assumption was verified using log-minus-log plots and time-dependent covariates, where applicable.

All statistical analyses were performed using SPSS Statistics (IBM Corp., Armonk, NY, USA, Version 29.0.1.0 (171)). The initial collection of patient data was carried out using Microsoft Excel (Microsoft Corporation, Redmond, WA, USA, Version 16.16.27 (201012)). A *p*-value < 0.05 was considered statistically significant.

Additionally, a literature review was conducted, and the findings were discussed in the context of our results. The following search terms were used: “serum ferritin,” “resection,” and “intrahepatic cholangiocarcinoma.” The electronic databases included PubMed, Google Scholar, and MEDLINE.

## Results

A total of 95 patients were included in the analysis. The mean age at the time of diagnosis was 64 years (range: 37–81), and the mean age at the time of resection was 65 years (range: 38–81). Fifty patients (52.6%) were male, and 45 patients (47.4%) were female. Patient characteristics are summarized in Table 1.

**Table 1:** Characteristics of patients

Variables	Entire Cohort (*n* = 95)
Age at the time of first diagnosis (years, mean, range)	64 (37– 81)
Age at the time of resection (years, mean, range)	65 (38–81)
Gender (Male, Female, n (%))	50 (52.6), 45 (47.4)
BMI (kg/m^2,^ mean, range)	26.3 (18–39)
Liver disease (yes, n (%))	
Steatosis	39 (41.1)
Fibrosis	8 (8.4)
Cirrhosis	4 (4.2)
Operation time (minutes, mean, range)	229.4 (85–437)
G Stage (n (%))	
G1 and G2	52 (54.7)
G3	43 (45.3)
N Stage (n (%))	
N1/Nx	43 (47.8)
N0	47 (52.2)
T Stage (n (%))	
T1	39 (41.5)
T2	39 (41.5)
T3	11 (11.7)
T4	5 (5.3)
Tumor size (cm, mean, range)	7.1 (1.7–19.5)
Tumor size (n (%))	
≥ 5 cm	70 (74.5)
< 5 cm	24 (25.5)
Tumor localization (n (%))	
bilobar	11 (11.6)
right lobe	39 (41.1)
left lobe	37 (38.9)
central	8 (8.4)
Tumor lesions (n (%))	
1	55 (57.9)
2	11 (11.6)
3	8 (8.4)
multiple	21 (22.1)
CA 19 − 9 highest (U/ml)	5825.69 (8.6–278739)
CA19-9 (n (%))	
elevated	64 (67.4)
normal	31 (32.6)
Albumin (g/L, mean, range)	36.22 (19–63)
Albumin	
> 30 g/L	75 (79.8)
≤ 30 g/L	19 (20.2)
Ferritin (µg/L, mean, range)	764.70 (24-12107)

### Prognostic cut-off value for SF

The optimal preoperative SF cut-off value for overall survival was determined using ROC analysis.

**Fig. 1:**
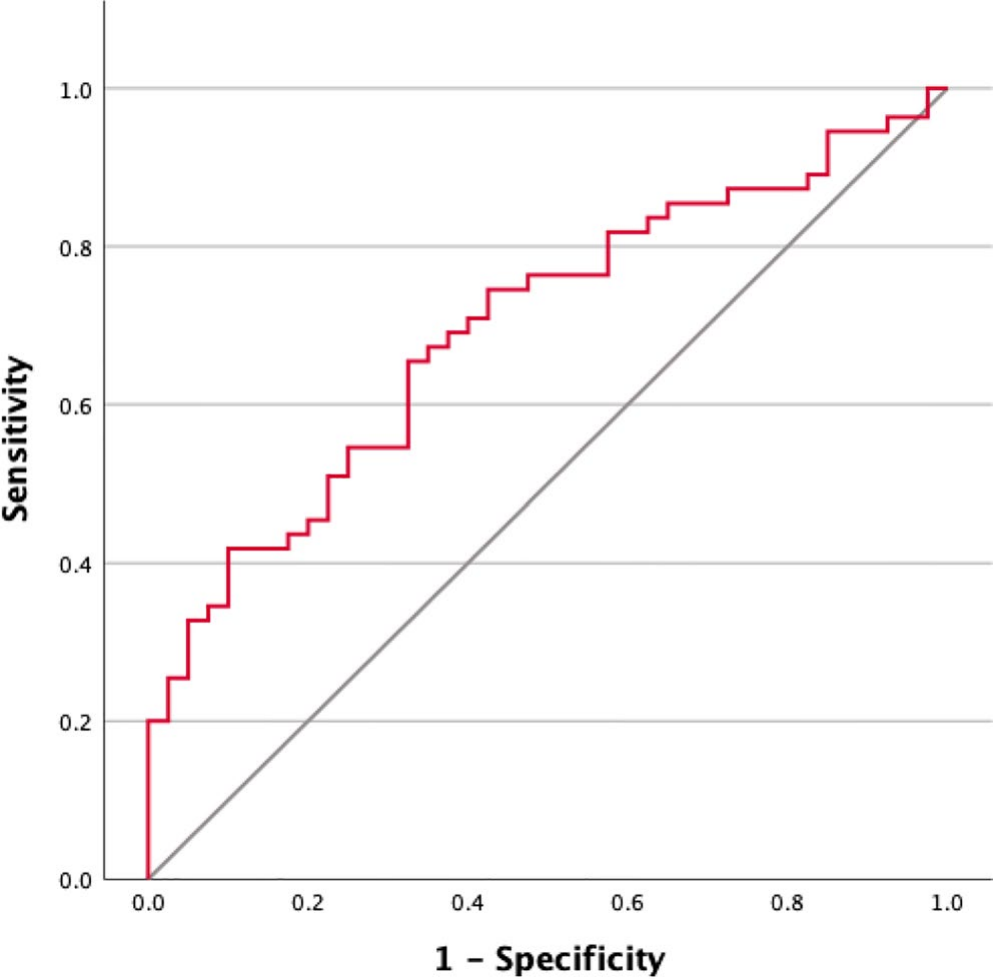
Receiver operating characteristics curves to evaluate the optimal cut-off value of serum ferritin for overall survival

The best cut-off value for SF was identified as 303.1 µg/L, with an area under the curve (AUC) of 0.697, a 95% confidence interval (CI) ranging from 0.592 to 0.801 (*P* < 0.001), a sensitivity of 65.5%, and a specificity of 67.5% (Fig. 1). Based on this threshold, patients were stratified into either a low-SF group or a high-SF group for further analysis. A comparison of patient characteristics between the high and low serum ferritin groups is presented in Table 2.

**Table 2:** Comparison of patient characteristics between the high and low serum ferritin groups

	High SF group (> 303.1 µg/L)	Low SF group (≤ 303.1 µg/L)
Total number (n(%))	49 (51.6)	46 (48.4)
Age at the time of first diagnosis (years, mean, range)	64 (38–81)	66 (37–80)
Age at the time of resection (years, mean, range)	64 (38–81)	66 (38–80)
Gender (Male, Female, n)	Male: 27	Male: 23
	Female: 22	Female: 23
BMI (kg/m^2,^ mean, range)	25.9 (18–34)	26.7 (18–39)
Liver disease (yes)		
Steatosis	20	19
Fibrosis	5	3
Cirrhosis	1	3
Operation time (minutes, mean, range)	245 (85–437)	213 (88–371)
Small for sized		
Yes	7	2
No	42	44
G Stage (n)		
G1 and G2	25	27
G3	24	19
N Stage (n)		
N1/Nx	29	14
N0	18	29
T Stage (n)		
T1	16	23
T2	21	18
T3	9	2
T4	3	2
Tumor size (cm, mean, range)	7.7 (1.7–19.5)	6.48 (2.5–13)
Tumor size (n)		
≥ 5 cm	41	29
< 5 cm	8	16
Tumor localization (n)		
bilobar	7	4
right lobe	18	21
left lobe	18	19
central	6	2
Tumor lesions (n)		
1	23	32
2	8	3
3	4	4
multiple	14	7
CA 19 − 9 highest (U/ml)	8623.6 (8.9-278739)	2845.315 (8.6-99731)
CA19-9 (n)		
elevated	34	30
normal	15	16
Albumin (g/L, mean, range)	34.1 (19–47)	38.37 (26–63)
Albumin		
> 30 g/L	31	44
≤ 30 g/L	17	2
Ferritin (µg/L, mean, range)	1333.69 (207-12107)	158.60 (24–299)
Relapse		
Yes	33	20
No	16	26
Overall Survival		
alive	13	27
deceased	36	19

### Survival analysis

#### Total cohort

The mean follow-up time from the date of resection was 32.6 months (range: 0.03–151 months). A total of 55 patients had died during the follow-up period, resulting in an OS rate of 42.1%. The 1-, 3-, and 5-year survival rates were 74.7%, 50.5%, and 43.2%, respectively.

A positive lymph node status (*P* = 0.010), poor tumor differentiation (*P* = 0.040), advanced T stage (*P* = 0.001), elevated preoperative serum ferritin levels (*P* < 0.001), decreased albumin levels (≤ 30 g/L) (*P* = 0.013), and increased tumor size (*P* = 0.016) were all significantly correlated with poorer overall survival (Table 3).

**Table 3:** Results of the Kaplan-Meier analysis

Variables	Univariate Analysis			Multivariate Analysis		
	*N* (%)	OS	*P*-value	HR	95%CI	*P*-value
N-Stage (Fig 2a)			0.010	1.770	0.939– 3.336	0.077
N0	47 (52.2)	39.7%				
N1/Nx	43 (47.8)	21.4%				
N-Stage and SF (Fig 2b)			< 0.001			
Low SF and N0	29 (32.2)	56.3%				
Low SF and N1/Nx	14 (15.6)	44.9%				
High SF and N0	18 (20)	9.5%				
High SF and N1/Nx	29 (32.2)	7.2%				
G-Stage (Fig 3a)			0.040	1.398	0.744– 2.625	0.298
G1/G2	52 (54.7)	38.8%				
G3	43 (45.3)	16.8%				
G-Stage and SF (Fig 3b)			< 0.001			
High SF and G1/G2	25 (26.3)	8.2%				
High SF and G3	24 (25.3)	0%				
Low SF and G1/G2	27 (28.4)	60.6%				
Low SF and G3	19 (20)	32.4%				
T-Stage (Fig 4a )			0.001			0.026
T1	39 (41.5)	46.9%				
T2	39 (41.5)	20.6%				
T3	11 (11.7)	14.1%				
T4	5 (5.3)	0%				
T-Stage and SF (Fig 4b)			< 0.001			
Low SF and T1	23 (24.5)	62.1%				
High SF and T1	16 (17)	0%				
Low SF and T2	18 (19.1)	40.4%				
High SF and T2	21 (22.3)	0%				
Low SF and T3	2 (2.1)	50%				
High SF and T3	9 (9.6)	13.3%				
Low SF and T4	2 (2.1)	50%				
High SF and T4	3 (3.2)	0%				
Preoperative SF (Fig 5)			< 0.001	2.849	1.408– 5.762	0.004
Low SF (≤ 303.1 µg/L)	46 (48.4)	50.9%				
High SF (> 303.1 µg/L)	49 (51.6)	4.5%				
Preoperative Albumin (Fig 6a)			0.013	1.094	0.538– 2.227	0.804
Albumin (> 30 g/L)	75 (79.8)	33.5%				
Albumin (≤ 30 g/L)	19 (20.2)	9.9%				
Preoperative Albumin and SF (Fig 6b)			< 0.001			
High SF and Albumin (> 30 g/L)	31 (33)	0%				
High SF and Albumin (≤ 30 g/L)	17 (18.1)	6.4%				
Low SF and Albumin (≤ 30 g/L)	2 (2.1)	100%				
Low SF and Albumin (> 30 g/L)	44 (46.8)	49.1%				
Tumor size (Fig 7a)			0.016	0.679	0.288–1.600	0.376
≥ 5 cm	70 (74.5)	20.7%				
< 5 cm	24 (25.5)	59%				
Tumor size and SF (Fig 7b)			< 0.001			
Low SF and tumor < 5 cm	16 (17)	76%				
Low SF and tumor ≥ 5 cm	29 (30.9)	40.4%				
High SF and tumor < 5 cm	8 (8.5)	43.8%				
High SF and tumor ≥ 5 cm	41 (43.6)	4.8%				
CA 19 − 9			0.072	0.908	0.469– 1.756	0.774
High CA 19 − 9	64 (67.4)	21.3%				
Normal CA 19 − 9	31 (32.6)	45%				
CA 19 − 9 and SF (Fig 8)			< 0.001			
Low SF and normal CA 19 − 9	16 (16.8)	67%				
Low SF and high CA 19 − 9	30 (31.6)	40.6%				
High SF and normal CA 19 − 9	15 (15.8)	0%				
High SF and high CA 19 − 9	34 (35.8)	4.5%				

### High and low SF

**Fig. 2:**
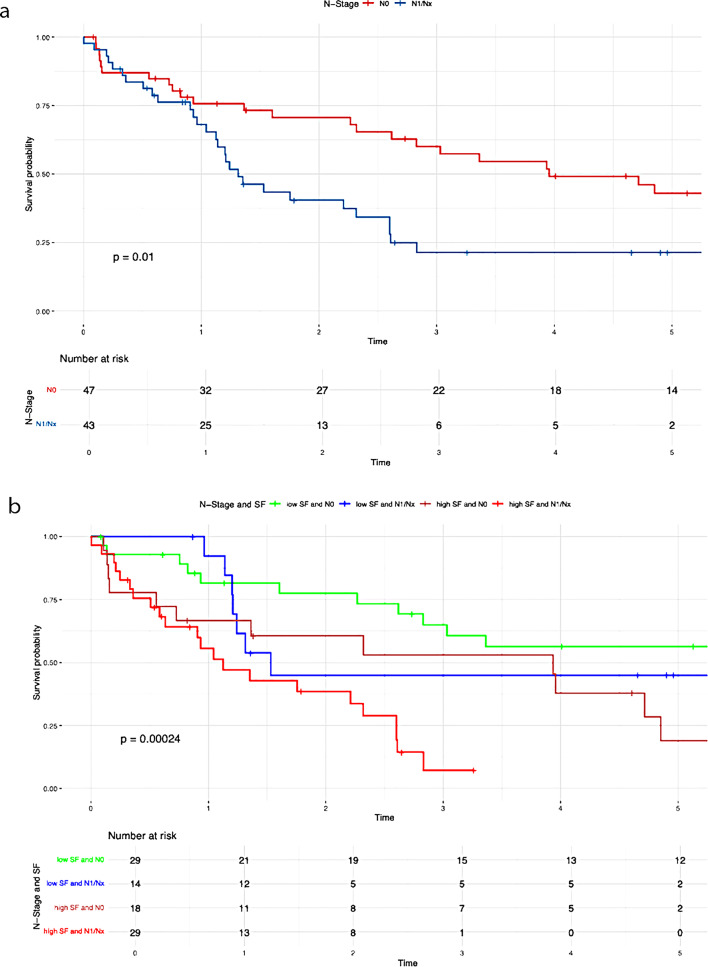
Overall survival N-Stage. (**a**) N-Stage (**b**) N-Stage and SF

Patients with elevated preoperative SF levels demonstrated significantly worse overall survival compared to the low SF group (50.9% vs. 4.5%; *P* < 0.001) (Table 3; Fig. 2).

**Fig. 3:**
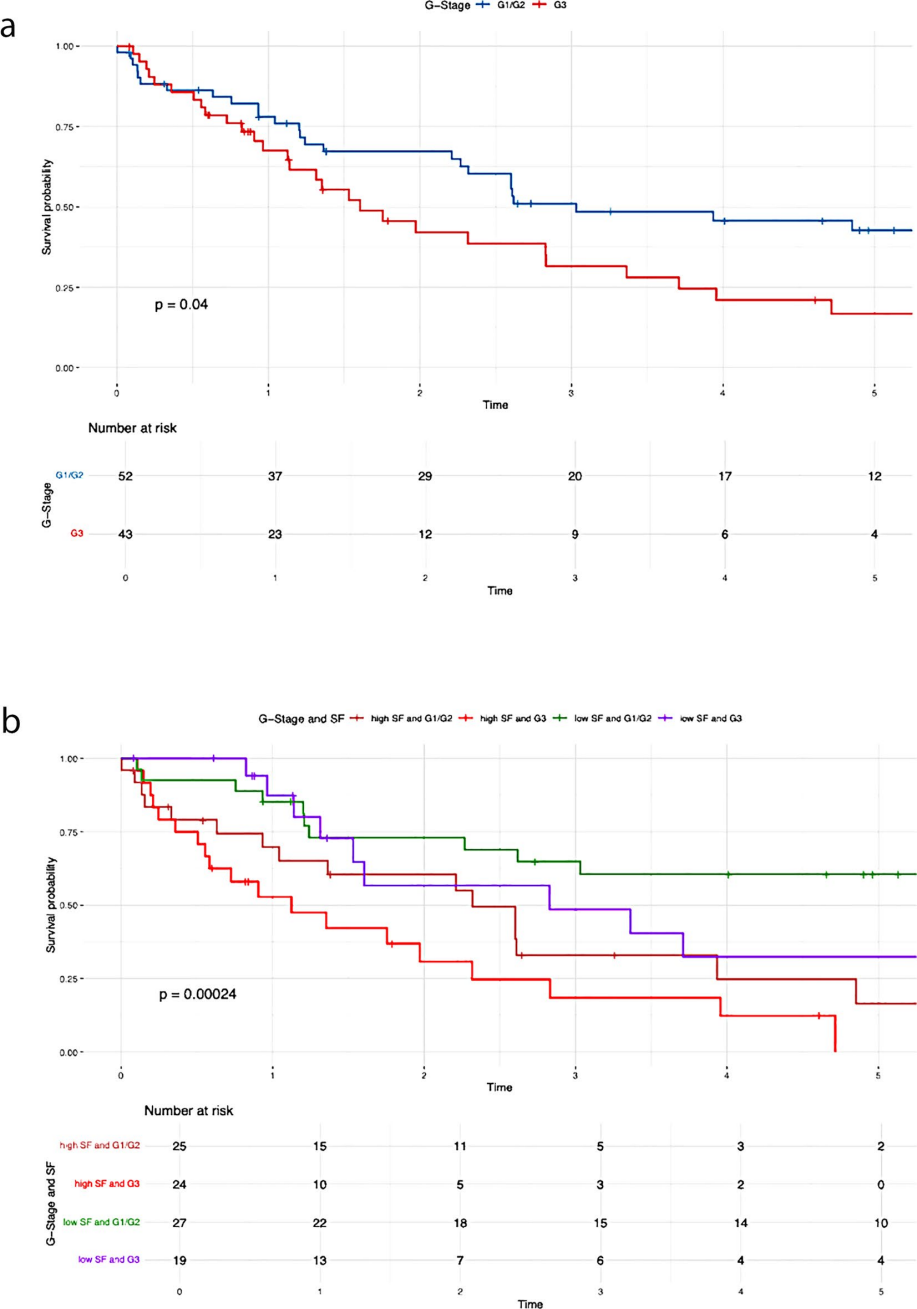
Overall survival G-Stage. (**a**) G-Stage (**b**) G-Stage and SF

**Fig. 4:**
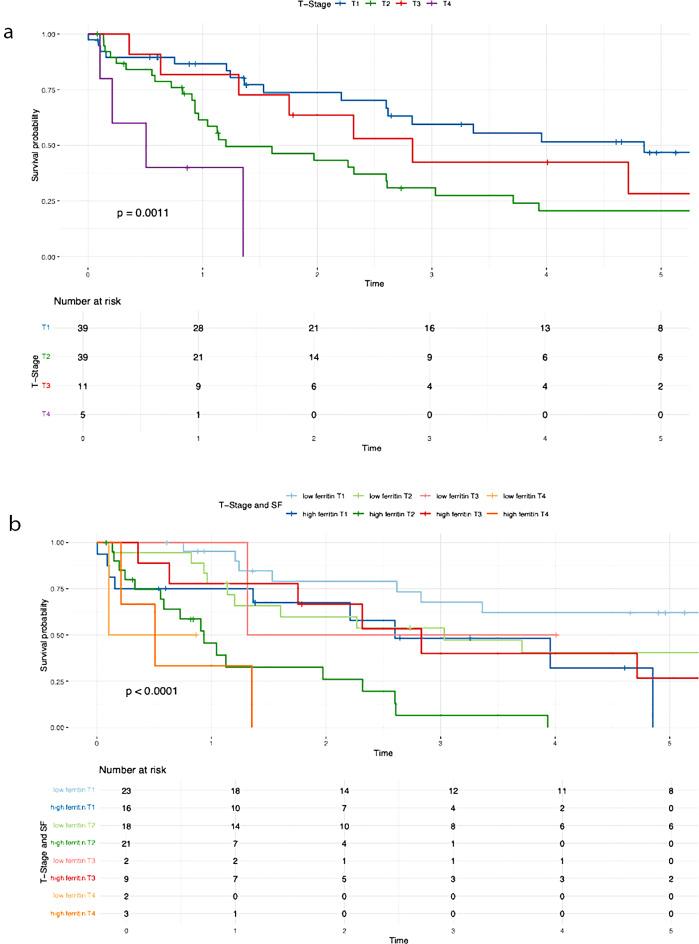
Overall survival T-Stage. (**a**) T-Stage (**b**) T-Stage and SF

Patients with elevated serum ferritin levels and positive lymph node status demonstrated significantly poorer overall survival compared to those with low serum ferritin levels (P = < 0.001) (Fig. 2b). The negative impact of elevated SF was also evident in relation to the following variables: G-stage (P = < 0.001) (Fig. 3b ), T-stage (P = < 0.001) (Fig. 4b), preoperative albumin levels (*P* = 0.013) (Fig. 6b), tumor size (P = < 0.001) (Fig. 7b), and CA 19 − 9 levels (P = < 0.001) (Fig. 8). The results of the Kaplan-Meier analysis are presented in Table 3.

### Multivariate analysis

Of the total 95 patients, 88 (92.6%) were included in the multivariable Cox regression model (Table 3). Seven patients were excluded due to missing values in one or more covariates.

In the multivariate Cox regression analysis including ferritin, T-stage, G-stage, tumor size, N-stage, albumin, and CA19-9, elevated preoperative serum ferritin remained a significant independent prognostic factor for overall survival (HR = 2.85; 95% CI: 1.41–5.76; *p* = 0.004). T-stage also demonstrated a statistically significant association with survival (*p* = 0.026), particularly T-stage 1 (HR = 2.16; *p* = 0.025). Although T-stage 3 showed a higher hazard ratio (HR = 2.76), it did not reach statistical significance (*p* = 0.120). N-stage showed a trend toward significance (HR = 1.77; *p* = 0.077), while G-stage, tumor size, albumin, and CA19-9 were not significantly associated with overall survival (*p* > 0.05 for all).

### Relapse rate

By the final follow-up date, 53 patients (55.8%) experienced confirmed recurrence of iCCA. The 1-, 3-, and 5-year recurrence-free survival rates were 55.8%, 46.3%, and 44.2%, respectively. Intrahepatic recurrences were the most frequently observed.

Among patients with elevated serum ferritin levels (> 303.1 µg/L), 33 of 49 (67.3%) developed recurrence. In the low-SF group (≤ 303.1 µg/L), recurrence was observed in 20 of 46 patients (43.5%). The difference in recurrence rates between the two groups was statistically significant (*P* < 0.001) (Table 4).

**Table 4:** Recurrence rates

Group	Total	Recurrence	Rate percent
Low SF	46	20	43.5
High SF	49	33	67.5
Total	95	53	55.8

P-value: <0.001

## Discussion

**Fig. 5:**
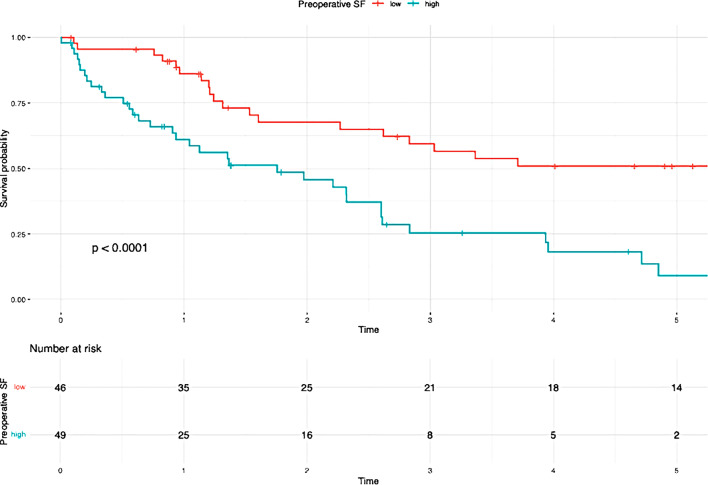
Overall survival Preoperative SF

In our study, we were able to demonstrate a significant association between elevated preoperative SF levels and overall survival (OS) as well as recurrence rates in patients with iCCA. The OS rate was 42.1%, with 1-, 3-, and 5-year survival rates of 74.7%, 50.5%, and 43.2%, respectively. Elevated preoperative SF levels were significantly associated with worse overall survival (low SF group: 50.9% vs. high SF group: 4.5%; *P* < 0.001) and higher recurrence rates (low SF group: 43,5% vs. high SF group: 67,3%; *P* < 0.001) (Fig. 5). In 1977, Hazard and Drysdale first reported a potential association between elevated SF levels and malignant diseases [7]. Since then, SF has increasingly gained attention as a potential biomarker, with numerous studies demonstrating a correlation between elevated SF levels and OS and recurrence rates in various malignancies, including neuroblastoma [13], glioblastoma [14], renal cell carcinoma [15], melanoma [16], non-small cell lung cancer [17], Hodgkin lymphoma [18], pancreatic cancer [9, 19, 20], breast cancer [21], colorectal carcinoma [12], and hepatocellular carcinoma [22–24]. Kalousová et al. demonstrated in their study that elevated SF levels are associated with poorer prognosis in patients with pancreatic cancer. Their analysis of 57 patients revealed that SF is a significant independent predictor of mortality in both univariate (*P* < 0.001) and multivariate (*P* = 0.002) analyses [9]. A study published in 2010 by Kukulj et al. [17] investigated the relationship between iron and inflammatory markers and OS in 125 male patients with non-small cell lung cancer. Their findings revealed that more than half of the patients presented with significantly elevated SF levels at the time of diagnosis, while serum iron levels were below the reference range. The authors concluded that increased ferritin expression in tumor tissue and elevated SF levels are more likely indicative of a response to acute inflammation, oxidative stress, and localized toxicity within tumor tissue rather than systemic iron overload. The impact of SF has also been examined in primary liver tumors. Facciorusso et al. demonstrated that elevated SF levels are a negative prognostic factor for both OS and recurrence rates in patients with HCC undergoing percutaneous radiofrequency ablation [22]. Patients with elevated SF levels (> 244 ng/ml) exhibited significantly poorer survival (31 months) compared to patients with lower SF levels (≤ 244 ng/ml; 78 months; *P* < 0.001). Furthermore, elevated SF was significantly associated with reduced recurrence-free survival (55 months vs. 15 months; *P* < 0.001). Similar findings were reported by Wu et al. [25]. Their 2019 study included 427 HCC patients who underwent curative liver resection for hepatocellular carcinoma. The optimal SF cut-off value for OS was identified as 267 ng/mL. The analysis demonstrated that preoperative SF levels independently predicted both OS and recurrence-free survival, regardless of other prognostic factors. Patients with lower SF levels exhibited better OS (*P* = 0.001) and RFS (*P* < 0.001). The 5-year OS was 45.2% for patients with low SF compared to 29% for those with high SF. Although the impact of SF has been extensively studied in various malignancies, there is still limited research examining the association between elevated SF levels and OS or recurrence rates in patients with iCCA. Xun et al. conducted a retrospective analysis of prognostic risk factors in 104 iCCA patients. Their findings revealed that ferritin concentration was an independent risk factor for survival, as shown in both univariate and multivariate analyses. Patients with high preoperative ferritin levels had a 1.7-fold increased risk of mortality compared to those with normal ferritin levels [26]. Similarly, Ma et al. confirmed that ferritin serves as a predictor for both disease-free survival and OS in iCCA patients. They concluded that ferritin could complement CA19-9 in stratifying survival outcomes, particularly for patients with small-duct-type iCCA [27]. These findings are consistent with ours. Although CA19-9 alone did not have a statistically significant impact on OS in our analysis (*P* = 0.072), likely due to the small sample size, a significant correlation (*P* < 0.001) (Fig. 8) was observed when preoperative serum ferritin levels were included. The OS for patients with elevated SF and CA19-9 levels was 4.5%, compared to 67% for those with low SF and normal CA19-9 levels (Fig. 8). This supports the notion that preoperative SF may serve as a valuable complement to CA19-9 as a tumor marker. While SF has been identified as a prognostic risk factor by several authors, an optimal cut-off value remains undefined. Therefore, we calculated the best cut-off value for SF using ROC analysis. The optimal cut-off value was determined to be 303.1 µg/L, with a sensitivity of 65.5% and a specificity of 67.5% (Fig. 1). Similarly, Facciorusso et al. [22] and Wu et al. [25] identified optimal SF cut-off values of 244 ng/mL and 267 ng/mL, respectively. Although our cut-off value of 303.1 µg/L differs slightly, it is important to note that our study focused on patients with iCCA rather than HCC. This could explain the higher cut-off value identified in our study.

In our study, we were also able to demonstrate that an increase in preoperative SF is significantly associated with worse OS (50.9% vs. 4.5%; *P* < 0.001) and a higher recurrence rate (67,3% vs. 43.5%; *P* < 0.001).

Additionally, a correlation was observed between elevated SF concentrations and tumor size as well as tumor differentiation (Figs. 2b and 3b). The significant impact of tumor size on survival in patients with iCCA has been repeatedly demonstrated [28, 29]. Sapisochin even showed that patients with iCCA and a tumor diameter of less than 2 cm had excellent survival rates even after liver transplantation [30]. This correlation is also reflected in our results. The overall survival was 59% for patients with a tumor diameter < 5 cm, compared to 20.7% for those with a tumor diameter ≥ 5 cm (*P* = 0.016), as shown in Table 2. It is noteworthy that the overall survival of patients assigned to the low SF group was 76% (tumor diameter < 5 cm) and 40.4% (tumor diameter ≥ 5 cm). In contrast, in the case of high preoperative SF, the overall survival decreased to 43.8% (tumor diameter < 5 cm) and 4.8% (tumor diameter ≥ 5 cm) (*P* < 0.001). Both for surgical resections and in the context of liver transplantation, a correlation between poor tumor differentiation and high recurrence or low overall survival rates in iCCA patients has been demonstrated [31–33]. Our findings support these results. Patients with poor tumor differentiation (G3) showed a significantly worse OS (16.8%) compared to those with G1/G2 differentiation (38.8%) (*P* = 0.040). Interestingly, a significant decrease in OS was also observed for patients in the high SF group with a G1/G2 tumor (OS: 8.2%) (Fig. 3b). In cases of high preoperative SF and a G3 tumor, overall survival dropped to 0%. The exact pathological mechanism of SF remains unknown. However, it is known that elevated SF concentrations do not necessarily correlate with an increase in transaminases, suggesting that the rise in SF is not a response to liver cell damage [24]. Similarly, an increase in SF does not seem to reflect changes in the body’s iron stores [17, 34]. Tappin et al. suggested that the increase in SF is partially due to a local release in the tumor area, at least in patients with breast tumors. After surgical resection, the SF level decreased by about 50%, which underscores the connection between tumor mass and high SF [35]. Although the impact of SF on tumorigenesis is still not fully understood, there are indications that extracellular ferritin can directly enhance proliferation in cancer cells, increase angiogenesis, and suppress lymphocyte responses [36–38]. Overall, these effects could contribute to tumor development and thus provide a possible explanation for why ferritin secretion plays a direct role in promoting and maintaining tumor progression. Nonetheless, our study has several limitations. Firstly, as a retrospective study, it is subject to inherent biases that cannot be entirely eliminated. Furthermore, the sample size was limited, and data were obtained from a single center. As such, the results should be interpreted with caution and require validation through larger, multicenter studies to ensure broader applicability. Furthermore, no postoperative assessment of serum ferritin levels was performed. As a result, dynamic changes in serum ferritin could not be evaluated. This limitation may serve as the basis for future studies.

## Conclusion

In summary, elevated preoperative SF levels in patients with iCCA are associated with significantly worse OS and higher recurrence rates. The optimal cutoff value for SF was 303.1 µg/l, with a sensitivity of 65.5% and a specificity of 67.5%. Although our results are statistically significant, further studies are required to investigate the exact pathomechanism of SF.

**Fig. 6:**
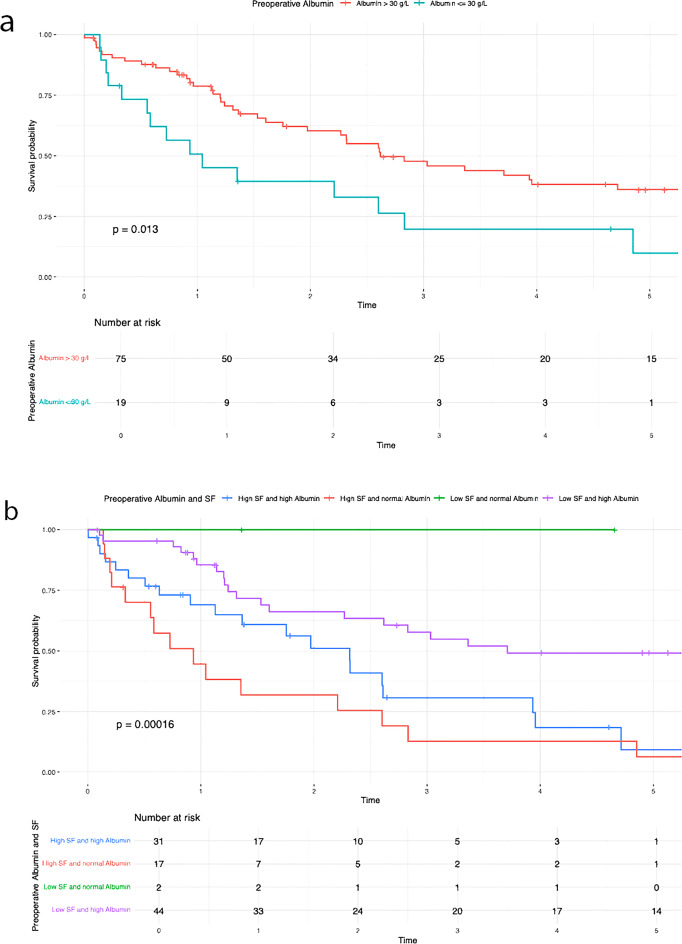
Overall survival preoperative Albumin. (**a**) preoperative Albumin (**b**) preoperative Albumin and SF

**Fig. 7:**
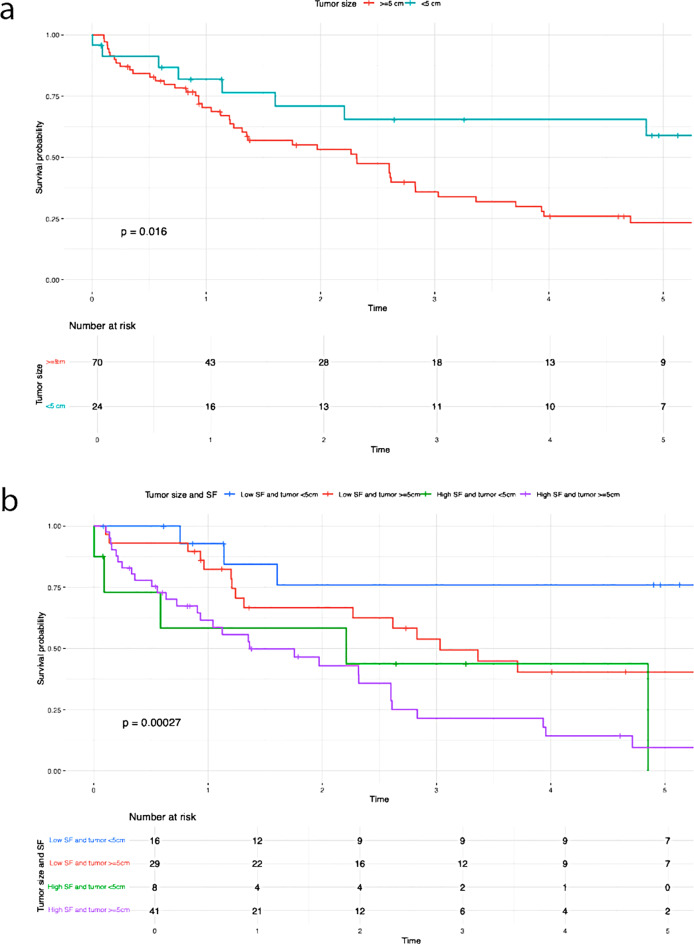
Overall survival Tumor size. (**a**) Tumor size (**b**) Tumor size and SF

**Fig. 8:**
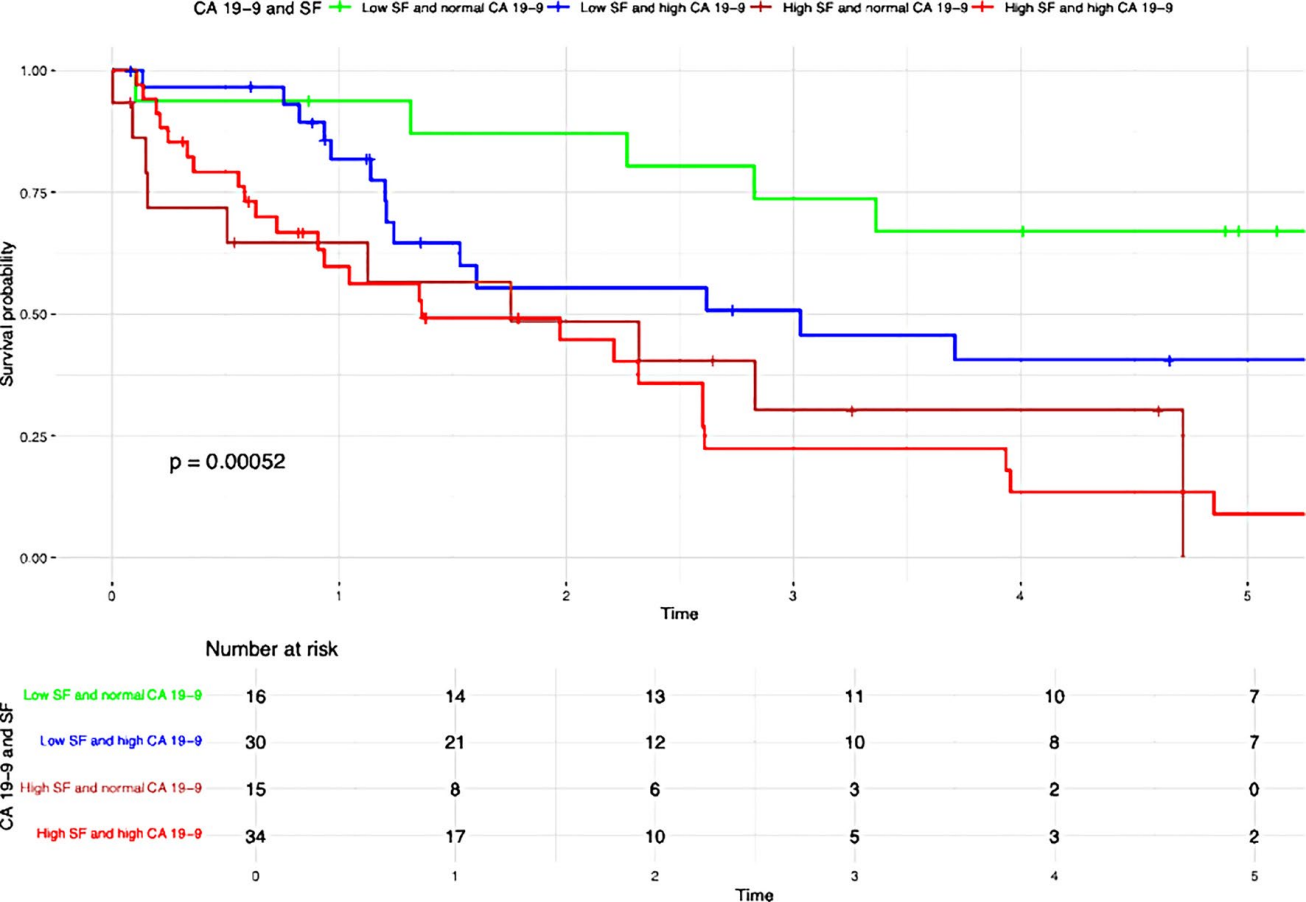
Overall survival CA19-9 and SF

## Data Availability

No datasets were generated or analysed during the current study.
